# Hyperthermia and Temperature-Sensitive Nanomaterials for Spatiotemporal Drug Delivery to Solid Tumors

**DOI:** 10.3390/pharmaceutics12111007

**Published:** 2020-10-22

**Authors:** Mohamadreza Amin, Wenqiu Huang, Ann L. B. Seynhaeve, Timo L. M. ten Hagen

**Affiliations:** 1 Laboratory of Experimental Oncology (LEO), Department of Pathology, Erasmus Medical Center, 3015GE Rotterdam, The Netherlands; m.amin@erasmusmc.nl (M.A.); w.huang@erasmusmc.nl (W.H.); a.seynhaeve@erasmusmc.nl (A.L.B.S.); 2 Nanomedicine Innovation Center Erasmus (NICE), Erasmus Medical Center, 3015GE Rotterdam, The Netherlands

**Keywords:** hyperthermia, temperature sensitive nanoparticles, liposomes, polymeric nanoparticles, triggered drug release

## Abstract

Nanotechnology has great capability in formulation, reduction of side effects, and enhancing pharmacokinetics of chemotherapeutics by designing stable or long circulating nano-carriers. However, effective drug delivery at the cellular level by means of such carriers is still unsatisfactory. One promising approach is using spatiotemporal drug release by means of nanoparticles with the capacity for content release triggered by internal or external stimuli. Among different stimuli, interests for application of external heat, hyperthermia, is growing. Advanced technology, ease of application and most importantly high level of control over applied heat, and as a result triggered release, and the adjuvant effect of hyperthermia in enhancing therapeutic response of chemotherapeutics, i.e., thermochemotherapy, make hyperthermia a great stimulus for triggered drug release. Therefore, a variety of temperature sensitive nano-carriers, lipid or/and polymeric based, have been fabricated and studied. Importantly, in order to achieve an efficient therapeutic outcome, and taking the advantages of thermochemotherapy into consideration, release characteristics from nano-carriers should fit with applicable clinical thermal setting. Here we introduce and discuss the application of the three most studied temperature sensitive nanoparticles with emphasis on release behavior and its importance regarding applicability and therapeutic potentials.

## 1. Introduction

For a chemotherapeutic drug to be effective the concentration reaching the target site (e.g., the tumor) needs to be sufficient enough to eradicate all tumor cells, which is one of the major challenges in chemotherapy. Often the high cytotoxic activity of a therapeutic compound observed in vitro is not matched after administration in animal models or patients. When injected into the bloodstream low-molecular weight chemotherapeutic agents exhibit short plasma half-lives due to distribution throughout the entire body, fast clearance, neutralization, or degradation. While the large volume of distribution and fast clearance rate negatively impact intratumoral drug concentrations and exposure time in the tumor tissue [1], toxic effect on normal tissues or excreting organs simply make dose increase not an option [2].

Encapsulating chemotherapeutic agents into a carrier not only enables administration of poor water soluble drugs [3,4,5] or preserves drugs from inactivation and degradation [6,7,8], it significantly reduces the exposure of normal tissues to chemotherapeutics by decreasing the distribution volume, which is under ideal circumstance a restriction to the blood pool, therefore lessening unwanted side effects on normal tissues. Modifications such as making nano-sized particles and PEGylation, which is the addition of polyethylene glycol to diminish recognition by macrophages, detract recognition of nanoparticle by the reticuloendothelial system (RES, i.e., macrophages) [9], and together with stable association of the encapsulated drug with the vehicle, grant a prolonged circulation of drugs in blood. In principle such modifications aim at taking advantage of pathological features of tumors where permeation into the tumor is enhanced due to fenestrated tumor vasculature and nanoparticles are retained inside tumors due to a lack of a functional lymphatic drainage; a mechanism known as enhanced permeation and retention (EPR) effect [10,11], and so far EPR-based passive targeting was the central paradigm in cancer nanomedicine. It has been shown that encapsulation of doxorubicin (DXR) in nano-sized PEGylated liposomes resulted in 250-fold reduced clearance rate and 60-fold reduced volume of distribution to 5.9 L compared to 365 L for free DXR when administered in a dose of 50 mg/m^2^ in humans [12], minifying the main dose-limiting cardiotoxicity associated with DXR treatment [13]. Meanwhile, compared to free DXR, higher accumulation of liposomal DXR in tumor has been achieved [14,15,16,17]. In this respect several clinically approved nanoparticles have been developed for treatment of cancer. However, the clinical success of these nanodrugs mostly relies on reduced side effect rather than improved antitumor efficacy.

In addition to features of a tumor that are in favor of drug delivery based on EPR, there are several barriers limiting a predictable and effective drug accumulation inside the tumor. Heterogeneous blood supply resulting in hyper-perfused and non-perfused areas prevents a homogenous drug distribution. Additionally, high interstitial fluid pressure and high levels of matrix proteins limit systemic administered drug escaping the blood flow and entering the tumor interstitium. Tumors tend to have an abundance of fibroblasts and infiltrating immune cells re-designed towards a pro-tumoral status. These cells also absorb drugs lowering the availability for malignant cells. Cytotoxic drug levels do not reach all cancer cells causing re-growth of the partially affected tumor. Moreover, heterogeneous drug delivery creates concentration gradients leading to regions with non-cytotoxic drug levels, which may cause drug resistance [18,19,20,21,22,23,24,25]. Therefore, manipulating the tumor microenvironment to increase perfusion, permeability, and extravasation, or even normalization of tumor vasculatures, were found to enhance drug delivery in tumors [26,27,28,29]. A dense extracellular matrix in a tumor not only correlates with a high interstitial fluid pressure but also prevents distribution of drugs into deeper regions by decreasing the diffusive or convective movements of drugs inside the tumor interstitial space [30]. This holds true for both free and encapsulated chemotherapeutics but becomes more significant for nanoparticles compared to free drug molecules as a result of a larger size. An effective EPR relies on a homogenous distribution and permeability of the tumor-associated vasculature, which is more than often not the case. Vessel growth in a tumor is very heterogeneous resulting in hyper-vascularized versus non-vascularized areas, mature versus immature (leaky) vessels, and a functional blood flow versus stasis. This results in a very heterogeneous blood supply, nanoparticle delivery, and extravasation [31,32]. Even extravasated nanoparticles remain predominantly in the perivascular region and only minimal amounts penetrate into the tumor interstitium further [25,33,34]. Tumor heterogeneity imposes significant limitations on effective delivery of nanoparticles to tumors and is not only observed spatially within a tumor [35,36], but between individual tumors [37] and is dependent on the size, location, and most importantly growth rate. Compared to model murine tumors, human tumors grow slower making this heterogeneity even more prominent. It seems that passive accumulation of nanoparticles in a tumor via the EPR effect is not that effective in human cancer and worth mentioning that the concept of enhanced permeation i.e., extravasation of nanoparticles through inter-endothelial gaps present in tumor blood vessels, is now under serious debate [38,39].

Next to challenges with delivery to tumor, cellular availability of drugs encapsulated in nanoparticles is another challenge. For an encapsulated drug to become biologically active it is important that the drug dissociates from the carrier. As stated earlier most of the nanoparticles are built to be stable during circulation in blood or are devised to be unrecognizable by reticuloendothelial system (RES) (coined as stealth), in order to ensure a long blood circulation, which is a prerequisite for tumor accumulation. However, upon entering the tumor interstitium that stability or stealthiness turns against effective delivery of drugs to cells where only free drug molecules can be functional. It could be said that in general content release has been sacrificed in the favor of nanoparticle stability, although release of the drug is essential to achieve a therapeutic response. When PEGylated liposomal doxorubicin (PLD) extravasates into the tumor interstitium minimal DXR release or interactions with cells is observed due to the rigid liposome membrane and presence of Polyethylene glycol molecules. As a result, it remains inside the tumor for a long period of time and DXR delivery to cells mainly takes place upon degradation of liposomes in a slow process over several days [31,40]. We showed that even the liposomes taken up by cells remain intact inside the endosomal/lysosomal system for several days [31,32,41]. Therefore, accumulation of nanoparticles in a tumor does not imply cellular availability of the content or that targeting of subcellular structures occurs [42].

One approach to enhance cellular delivery is by using ligand-mediated internalization, where nanoparticles decorated with ligands that interact with appropriate receptors on cells are being taken up by endocytosis. However, despite numerous studies none of the preclinically accepted ligand-modified nanocarriers have shown enough clinical benefit to reach the market. Despite ligand-modification of nanoparticles enhances internalization into cells, it has been shown that cellular level of drugs delivered into cells does not necessarily imply the availability of free drug molecules to affect the subcellular targets [41]. Moreover, all problems related to inefficient drug delivery to tumor through nanoparticles still exist since active targeting of tumor cells relies first on passive extravasation to reach the targeted cells. In addition, ligand modification negatively impacts on pharmacokinetics and distribution of nanoparticles; while ligand modification reduces circulation lifetime of nanoparticles, because of augmented clearance rate, which in turn decreases tumor accumulation [43,44,45], addition of an avidity to nanoparticles results in a prompt interaction with tumor cells soon after extravasation, limiting distribution and penetration of nanoparticles deeper into the tumor [41].

The crucial importance of delivering free drug to tumor cells prompted researchers to exploit pathological features of tumor microenvironment, including reduced pH [46,47], increased enzymatic [48] or redox [49] activities, and design nanoparticles releasing payload in response to these endogenous stimuli [50,51,52,53,54]. However, regions with the appropriate climate are heterogeneously distributed throughout the tumor, response to these stimuli is often slow and uncontrollable, and the small difference between normal and malignant tissue necessitates vehicles responsive to a narrow range of differences. Furthermore, delivery of such endogenous trigger-sensitive nanoparticles again relies on passive accumulation. In contrast to endogenous stimuli, exogenous stimuli such as heat, ultrasound [55,56,57], light [58,59], and radio frequency electromagnetic fields [60,61] provide a great spatiotemporal control over triggering the release from nanoparticles. Among these ultrasound [62,63] and mild hyperthermia [40,64,65] have an additional advantage to enhance tumor permeability and have been employed to improve tumor accumulation of nanomedicines.

Here we focus on the use of mild hyperthermia (e.g., 42 °C) in the steered delivery and release of coadministered nanoparticles. Thermosensitive nanoparticles can be constructed that are stable in the blood flow (i.e., at temperatures of 37 °C) and release their content when a higher temperature is applied at the tumor site.

## 2. Hyperthermia and Its Clinical Application

Hyperthermia in treatment of cancer is defined as raising the temperature of tumor locally or systemically by external means and it is now being regarded as the fifth modality of treatment, next to surgery, chemotherapy, radiotherapy, and immune-therapy.

The application of local or systemic hyperthermia for cancer therapy goes back to around 3000 B.C when, for example, the so-called “fire drills” (hot blades and sticks) were being used for the treatment of breast cancer [66].

Application of local hyperthermia can roughly be divided into two strategies. Thermal ablation with temperature above 50 °C and mild hyperthermia using temperatures around 41–45 °C. The high temperature used in ablation irreversibly damages cellular structures, directly killing cells and destroying tissue and matrix components as well as the vasculature, leading to immediate tissue destruction. This is mostly suitable for organ confined tumors, such as hepatocellular carcinomas, however, as the tumor spreads in the surrounding tissue not all cells will be effectively ablated.

Mild hyperthermia can directly and indirectly affect tumor cells. Cells located in regions of the tumor with poor blood flow have higher sensitivity to heat due to the hypoxic and acidic condition and could be selectively killed by application of this type of heat [67,68,69]. Due to mild hyperthermia cell membrane permeability and tumor perfusion are increased and capability of repairing DNA damages is disturbed [70]. These, on the one hand result in greater drug delivery to tumor and cells, and on the other hand increase the sensitivity of cells to chemotherapeutics and radiation, boosting the effect of chemotherapy and radiotherapy when combined with hyperthermia [71,72]. In addition, mild hyperthermia can induce or improve antitumor activity [73,74,75,76]. For example, many of the heat-shock proteins (HSPs) released from heat-stressed cancer cells can activate antigen-presenting cells (APCs) [77,78,79].

A standard chemotherapy protocol, including doxorubicin, in combination with regional mild hyperthermia has been shown to improve significantly the short-time and long-time survival of soft tissue sarcoma patients as compared to chemotherapy alone [80,81], most likely because of altered cellular processes, including DNA repair, change in the tumor microenvironment, and anticancer immunity induced by hyperthermia [82].

**Figure 1:**
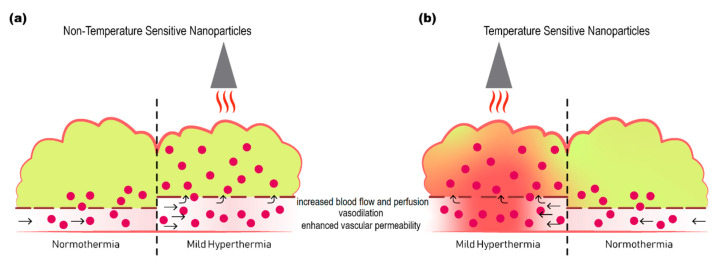
Schematic representation of the effect of mild hyperthermia on drug delivery to tumors. Delivery of non-temperature sensitive nanoparticles (**a**), or temperature sensitive nanoparticles (**b**) to tumor is enhanced by application of mild hyperthermia. Mild hyperthermia increases regional blood flow and perfusion, causes vasodilation, and increases vascular permeability, resulting in increased extravasation and more homogeneous distribution of nanoparticles deeper into the tumor. When combined with temperature sensitive nanoparticles the additional advantage is increased cellular delivery of free drug upon release from the nanoparticles in response to heat.

In addition to these effects mild hyperthermia also increases the permeability of tumor vasculature by enlarging pore size in the endothelial lining, facilitating nanoparticle extravasation, which, alongside increased perfusion, improves accumulation of liposomes [83,84,85] or free drug [86,87] inside the tumor interstitium (Figure 1). Enlargement of gaps in the endothelial lining is specific for the tumor-associated vasculature and is reversible after approximately 8 h [64].

Delivery of chemotherapeutic agents in a spatiotemporal controlled manner has long been a dilemma in the field of drug delivery. We believe that with good knowledge of the biology, and the unique features of the tumor tissue subjected to heat, and the advanced methods and instruments that enabled precisely controlled application of hyperthermia at desired regions, heat triggered drug release from temperature sensitive nano-devices is the most advanced and promising strategy to fulfill the concept of the magic bullet. However, the rate and magnitude of release from such nanoparticles are crucial with respect to efficient drug delivery. Herein we discuss the advances made with designing three main classes of temperature sensitive nanoparticles.

## 3. Thermosensitive Liposomes (TSLs)

Liposomes are spherical vesicles of one or multi layers of lipid bilayer phase, mainly composed of phospholipids, and spontaneously formed by self-assembly of amphipathic lipids upon exposure to aqueous environment. Unique features such as ease of preparation and modification, biocompatibility, biodegradability, capability of carrying variety of compounds either inside the aqueous core or inside the hydrophobic part of the membrane or on the surface of liposomes make them powerful tools for drug delivery. In addition, the advent of remote loading methods by which weak acidic or weak basic drug molecules could be encapsulated in liposomes at high concentration with high encapsulation efficiency, together with the high captured volume inside liposomes which provides ample space for encapsulation of hydrophilic molecules, allows efficient delivery.

**Figure 2:**
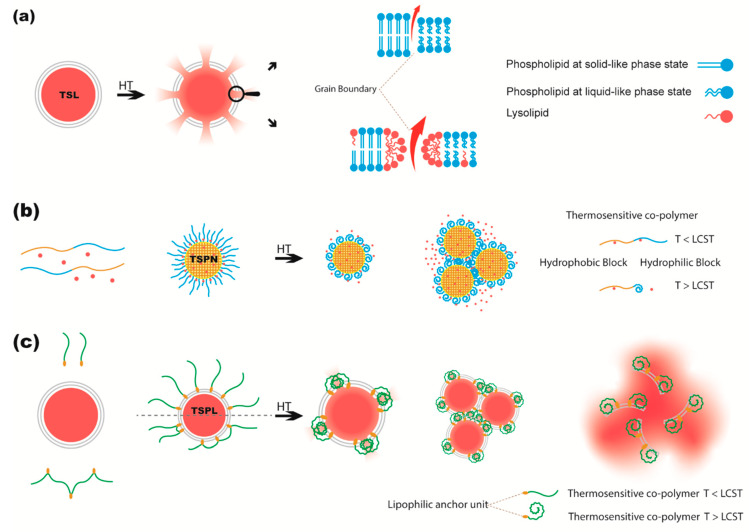
Schematic representation of postulated mechanisms of drug release from different temperature sensitive nanoparticles in response to exposure to hyperthermia (HT). (**a**) Upon heating of a temperature sensitive liposome (TSL) phospholipids grain boundaries form between coexisting phospholipids of solid-like and liquid-like states from which encapsulated drug releases. Presence of lysolipids stabilizes the grain boundaries leading to ultrafast drug release from the liposome at hyperthermia condition. (**b**) At temperatures (T) below the release temperature (lower critical solution temperature, LCST) of temperature sensitive copolymers, from which temperature sensitive polymeric nanoparticles (TSPN) have been formed, the outer shell of the nanoparticle consists of fully hydrated and stretched polymers. By increasing the temperature higher, the hydrated outer shell collapses by losing hydrophilicity and becomes hydrophobic which leads to drug release and aggregation of TSPN. (**c**) Upon heating of liposomes modified with temperature sensitive polymers (TSPL) to temperatures above the LCST, the temperature sensitive motifs of copolymers lose hydrophilic reactions and tend to interact with hydrophobic regions of phospholipid bilayers which results in drug release, aggregation, or disintegration of liposomes.

A unique feature of phospholipid bilayers is the ability to undergo a reversible thermotropic gel-to-liquid crystalline phase transition (Tc). In the gel state, the hydrocarbon chains of phospholipids are fully extended, packed closely side-by-side, and aligned almost perpendicular to the liposomes surface plain. As a result, phospholipids occupy the minimal cross-sectional area, and thickness of the bilayer is at a maximum (5.0–5.5 nm), depending of the length of acyl chain. Such highly ordered packing restricts intra- and intermolecular motions establishing a highly impermeable membrane. In the liquid crystalline state the acyl chains are more mobile towards the terminus of the acyl chain in the center of the bilayer, and flap around and tilt more at the glycerol backbone, resulting in increased effective cross-sectional area of phospholipid molecules and reduction of the bilayer thickness to about 4.0–4.5 nm. Consequently, membranes at the liquid crystalline phase are typically more permeable [88,89]. However, the highest permeability or release from liposomes is observed during this transition. Papahadjopoulos et al. found that self-diffusion rates of 22Na^+^ or 14C-sucrose through liposomes composed of DPPG or DPPC increase by increasing temperature and reach a maximum at temperatures close to transition temperature of the lipids, but after that release decreases with further increasing temperature [90,91]. The most compelling explanation for that, which is still accepted as the mechanism of release from temperature sensitive liposomes (TSLs), was suggested to be attributed to a transient formation of microscopic regions of disorder, later called “grain boundaries”, between domains of phospholipids in gel phase and domains of phospholipids in liquid phase that coexist within a membrane plane [90] (Figure 2a). Followed by increasing the temperature fraction of such disordered boundaries reduces as the membrane is predominantly in a liquid-like phase. In addition, when transition behavior of liposomes was abolished by addition of 50 mol% cholesterol the release was also completely attenuated. Evidence of coexisting clusters of gel and liquid crystalline phases have been observed in liposomes composed of DOPC and DSPC [92]. Considering these boundaries as the releasing sites in liposome membranes was also fit and predicted by mathematical modeling [90].

This property of release at the transition temperature was for the first time exploited by Yatvin and coworkers [93] to design liposomes with transition temperatures above 37 °C but attainable by mild hyperthermia, using DPPC-based liposomes. Liposomes composed of pure DPPC start to release encapsulated CF at around 30 °C and reach a maximum release at ca. 38 °C. Addition of 25 mol% DSPC to DPPC results in a sharper release at elevated temperatures. Onset temperature of release increases to ca. 41 °C, and maximum release was achieved at 45 °C, and prephase transition behavior observed in pure DPPC was abolished. It was also found that presence of serum is in favor of release by enhancing the magnitude of release without changing release temperature. Since then DPPC:DSPC mixtures were employed for making thermosensitive liposomes (TSLs). Later it was observed that injection of TSLs composed of DPPC:DSPC (70:30 mol%) encapsulating Methotrexate (MTX) into mice bearing a Lewis lung carcinoma resulted in almost three times higher levels of MTX 4 h after injection when the tumor was heated at 42 °C for 15 min before and 1 h after injection compared to unheated tumors [91,94,95]. The therapeutic benefit of TSLs containing cis-Dichlorodiammineplatinum(ll) was then evaluated in mice bearing sarcoma 180 tumors. Injection of liposomes plus mild hyperthermia applied as above resulted in greater drug uptake and longer tumor growth delay following treatment, which was equal to treatment with 2.5-fold higher dose of free drug plus mild hyperthermia [95]. However, the release rates from these liposomes is relatively slow [93,94,95]. Iga et al. [96] found that decreasing DSPC to 10 mol% resulted in faster and higher release compared to formulations with higher DSPC content. It was also found that the release is also affected by liposome size as larger liposomes are more sensitive to heat compared to smaller liposomes.

In addition to release properties the studied TSLs revealed fast clearance rates [94,95]. Maruyama et al. [97] increased the circulation time of TSLs by addition of 6 mol% ganglioside GM1 (GM1) to DPPC:DSPC (90:10) lipid mixture which resulted in 2.5 times increased tumor accumulation compared to TSLs without GM1. However, their TSLs released 45% of the loaded DXR after 5 min incubation at 42 °C in 20% serum. As stated earlier, addition of 50 mol% cholesterol into liposomes abolishes phase transition behavior and improves leakage stability of liposomes. In fact, the leakage stability of Doxil, a clinically approved form of liposomal doxorubicin, is partly attributed to the presence of 40% cholesterol in the lipid membrane. However, at lower contents of cholesterol endothermic events occur, but at lower extent negatively correlating with cholesterol content. Based on this Papahadjopoulos and colleagues employed cholesterol at low content and PEGylation to increase stability of TSLs while preserving the thermal responsiveness [98]. Their optimized TSLs composed of DPPC:HSPC:Chol:mPEG2000-DSPE (54:27:16:3 mol%) showed minimal leakage during 30 min incubation at 37 °C in presence of 50% bovine serum, whereas TSLs composed of DPPC:HSPC (67:33 mol%) released 50% of DXR content at this condition. However, addition of cholesterol negatively reduces both release rate and the magnitude of release at 42 °C where TSLs containing cholesterol and PEG releases about 50% over 30 min, while liposomes lacking cholesterol and PEG exhibit a maximum release of 70% within 10 min. It was found that when 1 h hyperthermia of 42 or 45 °C is applied 1 h after injection TSLs extravasate into interstitial space at the same extent as non-TSLs do, however, using TSLs resulted in 38 and 76-times higher levels of free DXR or CF inside the tumor interstitium upon 1 h hyperthermia at 42 or 45 °C, respectively [99]. We have shown that for thermoresponsive liposomes a concentration of 5 mol% mPEG2000-DSPE is an optimal concentration [100,101], which not only prolongs circulation time of TSLs in blood but also it is believed that the PEG molecules stabilize grain boundaries and facilitate DXR release [102]. We showed that TSLs composed of DPPC:DSPC:mPEG2000-DSPE (80:15:5) quickly release over 70% of encapsulated CF at 42 °C in presence of 100% serum, while less than 8% release was observed during 1 h incubation at 37 °C in serum. Using intravital imaging we found more than 30-fold increased DXR level in the heated tumor area compared to free DXR [100,101]. Interestingly, this preparation was found to be more effective in treatment of a mouse B16BL6 tumor compared to lysolipid containing TSLs [100].

As discussed earlier one of the goals of triggered drug release is to reduce the reliance of drug delivery on extravasation. To fulfill this, thermosensitive release needs to take place during the short transit time of the liposomes while traveling through the heated area, demanding sharp, fast, and high content release [103,104]. Drug molecules released inside the blood while passing through the heated area, i.e., the tumor, have a greater chance to extravasate and distribute inside the tumor. It has been found that intravascular DXR release from liposomes provides deeper penetration and more homogeneous DXR distribution compared to administration of free doxorubicin [103]. In addition to that, intravascular release results in exposure of endothelial cells to high concentration of cytotoxic drug. Uptake of the cytotoxic drug, e.g., DXR, by endothelial cells results in vascular damage and indirectly boosts the treatment efficacy by depriving tumor cells from oxygen and nutrition supply [100] and, in part, contribute to the enhanced antitumor efficacy of TSLs plus hyperthermia [100,105,106].

The great leap in application of thermoresponsive nanoparticles against cancer happened when Needham’s group found that incorporation of the lysolipid MPPC drastically increases the rate and magnitude of drug release from DPPC liposomes [107]. The mechanism of this ultra-fast release is related to the tendency of lysolipids to form highly curved micelles. When the temperature approaches the transition temperature of the DPPC membrane, lysolipids find their preferred curvature at the developed grain boundaries in solid–liquid interfaces, and the increased lateral movement at elevated temperature enables them to migrate to the faceted grain structures forming lysolipid-lined nanopores in the lipid bilayer [102]. In fact, both PEG and lysolipids stabilize the grain boundaries resulting in a burst release from nanoporous defects in the lipid bilayer. Lysolipid containing TSLs (LTSLs) composed of DPPC:MPPC:mPEG2000-DSPE (90:10:4 mol ratio) revealed higher therapeutic efficacy compared to non-TSLs or cholesterol containing TSLs when a mild hyperthermia of 42 °C was applied on the tumor for 1 h right after injection, and proved to benefit intravascular drug release [108]. However, LTSLs containing MPPC start to release DXR at 36 °C [108]. Replacing MMPC with MSPC improved the release profile by increasing the onset temperature of release, resulting in more stable liposomes at 37 °C [109]. The outperformance of this formulation in preclinical settings resulted in clinical evaluation of LTSLs under the commercial name Thermodox^®^ [102,104], first against liver cancer in combination with radiofrequency ablation (RFA), and for recurrent breast cancer combined with microwave hyperthermia [110].

Inspired by the results obtained with lysolipids the group of Shyh-DarLi used Brij78, a surfactant of C18 conjugated to PEG20 via an ether link, to enhance drug release from DPPC liposomes. The optimized preparation composed of DPPC:Brij78 (96:4 mol%) and remotely loaded with DXR exhibited comparable thermoresponsive release as was observed with LTSLs [111]. Brij78 not only provides pore formation at grain boundaries to enhance release, the PEG moiety provides steric stabilization. Compared to LTSLs composed of DPPC:MSPC:mPEG2000-DSPE (86:10:4 mol%), liposomes of DPPC:Brij78 (96:4 mol%) exhibited similar pharmacokinetics but appeared more effective in an EMT-6 tumor model after a dose of 3 mg DXR/kg and 1 h hyperthermia of 43 °C [112]. Later the loading method was changed from pH gradient to Cu^2+^ gradient, which improves the pharmacokinetics by a 2.5-times slower clearance rate and 2-times more DXR delivery to the heated tumor compared to LTSLs [113]. However, it should be noted that the most efficient temperature at which Thermodox releases DXR is 41–42 °C and it has been shown that release at 43 °C is significantly suppressed by almost 50% [102,109]. Therefore, the lower DXR delivery of LTSLs compared to Brij-TSLs could partly be attributed to overheating of the tumor at 43 °C in these experiments.

Next to the LTSL another advanced TSL was developed by the Lindner group. They found that addition of 1.2-dipalmitoyl-sn-glycero-3-phosphoglyceroglycerol (DPPGOG or DPPG2, transition temperature 39.7 °C), a derivative of the naturally occurring DPPG, into TSLs composed of DPPC:DSPC:DPPGOG (50:20:30 mol%) results in prolongation of the circulation time of TSLs (t1/2 9.6 h in hamsters and t1/2 5.0 h in rats) and greatly increases the release rate of CF from TSLs in response to mild hyperthermia of 42 °C [114], which was comparable to the fast release from LTSL composed of DPPC:MPPC:mPEG2000-DSPE (90:10:4 mol ratio). Interestingly, Lindner and coworkers show that DPPGOG-TSLs retained encapsulated CF up to 10 h at 37 °C in the presence of 90% serum, whereas PEG-TSLs and MPPC-LTSLs became unstable after 6 and 3 h respectively, in the same condition. In addition, DPPGOG-TSLs exhibited 69.7 ± 1.4% and 72.8 ± 1.8% DXR release during 18 s at 42 and 43 °C, respectively [115]. However, this has not been compared with MSPC-containing LTSLs. In addition to lipid content, the other parameter that they considered was liposome size. Since they aimed at intravascular release they made liposomes of around 200 nm, which is suitable for long circulating liposome but probably less suitable for extravasation. Two times increase in the diameter of liposomes increases the liposome volume by 8, which means a 200 nm liposome is capable of carrying eight times more encapsulated drug compared to a 100 nm liposome, making it more efficient for intravascular drug release. In addition to preclinical therapeutic efficacy of DXR loaded DPPGOG-TSL + hyperthermia in mouse tumor models, it also showed promising safety and therapeutic benefit when used for treatment advanced feline soft tissue sarcoma [116].

## 4. Temperature Sensitive Polymeric Nanoparticles (TSPN)

Advanced polymerization techniques and the vast compositional space of polymer architectures have stimulated a growing number of studies within the field of responsive polymers. Polymers exhibit the most expansive responsiveness to a variety of chemical, physical, and biological stimuli, including temperature [117,118].

Thermoresponsiveness of polymers results from solubility changes by differing the ambient temperature [118]. Based on the response by increasing the temperature thermoresponsive polymers could be classified into two groups: (a) some thermosensitive polymers are soluble in aqueous medium but their solubility reduces above a critical temperature and are known as polymers exhibiting lower critical solution temperature (LCST). (b) In other thermoresponsive polymers the solubility is reduced below a critical temperature, exhibiting upper critical solution temperature (UCST). Although, both kind of polymers have been used for production of a variety of polymer based thermosensitive nanoparticles, studies on polymers with LCST behavior largely outnumber those on UCST polymers.

Followed by pioneering works of Heskins and Guillet [119] on poly-*N*-isopropylacrylamide (PNIPAAm), which shed light on the mechanisms underlying thermal behavior of PNIPAAm in aqueous solution or in general other LSCT polymers, this polymer became a backbone in designing thermoresponsive polymers for biomedical applications because it exhibits an LCST around 32 °C, close to physiological temperature [120].

Other polymers such as polyethylene glycol (PEG), poly(propylene glycol) (PPG) [121], poly(glycidyl methyl ether-co-glycidyl ethyl ether) (P(GME-co-GEE)), poly(Nvinylcaprolactam) (PNVCL) and biopolymers such as leucine zipper, collagen, Silk [122] and Elastin-like polypeptides (ELP) [123] have also been used for preparation of thermosensitive drug delivery systems.

In a solution of thermoresponsive polymers the ambient temperature determines how the polymer interacts with water molecules. Below the critical temperature (i.e., LCST), hydrophilic parts of the polymer makes hydrogen bonds with water, as for example in PNIPAAm, the oxygen and nitrogen atoms of amide bond are accepting hydrogen bonds, while the covalently attached hydrogen to the nitrogen atom is a donor of hydrogen bonds, keeping the polymer hydrated as an expanded coil. By increasing the temperature the hydrogen bonds between polymer and water that keep the polymer soluble become weak. Loss of the hydrophilicity imposes restructuring of water molecules surrounding the now hydrophobic polymer which is entropically unfavorable. To compensate this entropic loss of the system water molecules decrease the contact surface with hydrophobic polymers. Water phase out the polymer from the solution and polymer undergoes coil (swollen hydrated state) to globule (shrunken dehydrated state) transition [124,125].

Such transition is suitable to be exploited for triggering release in response to exposure of nanoparticles made of temperature sensitive polymers to heat (Figure 2b). However, the temperature range at which the particles respond to heat needs to be rationally adjusted. For example, the LCST of the primary and the most studied PNIPAAm homopolymer is around 32 °C, which is below physiological temperature [124,125]. As stated earlier, LCST response arises from breaking the hydrophilic interactions of polymer with water. Therefore, addition of hydrophilic moieties to PNIPAAm can simply increase the extent of hydrophilic interactions with water and elevate the LCST. In this regard, many studying groups copolymerized PNIPAAm with hydrophilic monomers such as acrylamide (AAm) in PNIPA-co-AAm [126], acrylic acid (AAc) in PNIPAm-AAc [127], or *N*-hydroxymethylacrylamide NHMAAM in *P*-(NIPAAm-co-NHMAAm) [128], and successfully achieved copolymers with higher LCST (Table 1). However, in such systems hyperthermia plays as nanoparticle stabilizer rather than release inducer [126].

In addition to the LCST, critical micellar concentration (CMC) is another parameter that governs the stability of polymeric micelles in an aqueous environment. When micelles are diluted below the CMC, disassembly into unimolecular amphiphiles occurs. Copolymerization with hydrophobic monomers, such as polystyrene (Pst) in PIPAAm-PSt [129], butylmethacrylate (PBMA) in PIPAAm-PBMA [130], poly-l-lactide in *P*-(IPAAm-co-DMAAm)-b-*P*-(d,l-lactide), and caprolactone (PCL) in *P*-(NIPAAm-co-NHMAAm)-b-PCL [128] enhanced the micellar formation and stability of the nanoparticle in aqueous media. In contrast to the addition of hydrophilic monomers that increases the LCST, copolymerization of PNIPAAm with hydrophobic blocks showed no [129,130,131] or minor [128] effect on the LCST.

By means of copolymerization, choosing the number of repeating units and the molar mass, variety of nanoparticles with desired LCST to sense and release their payload have been fabricated [118]. In a thermoresponsive polymeric nanoparticle the hydrophilic part of a polymer is responsible for the thermosensitivity and also provides a hydrophilic corona around the nanoparticle that enhances circulation life time. Meanwhile, the hydrophobic part determines formation and stability of the particle scaffold, that could be used for incorporation of hydrophobic therapeutics (Table 1). However, the rate of drug release is still the most challenging part that has not yet fulfilled the needs of hyperthermic release at the tumor site. LSCT-based thermoresponsive polymeric nanoparticles exhibit an initial burst release at temperatures below the LSCT and, as can be seen in Table 1, they require prolong duration of hyperthermia, few hours to days, to release a significant amount of their payload, which is impractical for clinical use. In addition, for many reported nanoparticles release at 37 °C is almost identical to what observed at mild hyperthermia. During phase transition, collapse of the soluble thermoresponsive corona and nanoparticle aggregation result in drug release. It should be taken into account that during the phase transition physicochemical characteristics of the hydrophobic core remains intact, thereby the nanoparticle scaffold, which is loaded with drug, does not directly contribute to the drug release, explaining the slow rate of drug release from these nanoparticles. Sun et al. found that increasing the hydrophobicity of the block-copolymer is in favor of doxorubicin (DXR) incorporation into nanoparticles, but at the same time it hampers DXR release from nanoparticles even at temperatures higher than the LCST [132]. In addition to that, formation of a dense shrunken corona, as a result of thermal transition, may also restrict the diffusion of drug from these nanoparticles. Zhang et al. [126] reported a slower release of docetaxel at 43 °C than at 37 °C, from thermally responsive nanohydrogel, implying the deflation of nanogel porosity and blockage of release. Even more sophisticated structures of supramolecular polymers such as DXR-loaded Cy-PPG nanogels [121], requires 3 h for 88% DXR release at 40 °C, or in DXR-loaded PNI-U-DPy micelles (LCST 34 °C) after 1 h at 37 °C only 10% of DXR was released [133].

Although the temperature triggered release rate from thermosensitive polymeric nanoparticles does not fit with required drug release rates during applicable hyperthermia treatments, these nanoparticles are successful in induction of thermal triggered cellular interaction and internalization due to increased surface hydrophobicity upon transition [134,135,136,137,138]. Therefore, higher tumor accumulation and higher intracellular internalization of thermoresponsive nanoparticles inside the tumor could be achieved by application of heat. It needs to be taken into account that if surface hydrophobization takes place inside the tumor vasculature, while the intravascular concentration of polymeric nanoparticle is high, it may result in massive aggregation, clotting, and blockage of tumor vessels.

## 5. Temperature Sensitive Liposomes Sensitized by Temperature Sensitive Polymers

Modification of liposome membranes with a temperature sensitive polymer (TSP) goes back to studies of Ringsdorf and coworkers [143], where they incorporated a random copolymer of NIPAM and *N*-[4-(1′-pyrenyl)-butyl]-*N*-n-octadecylacrylamide into a liposome membrane in order to create a model membrane simulating the dynamics of the cytoskeleton to an external stimulus. Later Hoffman [144] conjugated around 4 mol% of a phopspholipid into an NIPAM backbone and inserted the thermoresponsive polymer into liposomes, micelles, cell membranes, and hydrophobic interfaces in order to create a thermal control function. However, it was Kano and coworkers that first utilized temperature sensitive polymers to achieve triggered drug release from liposomes in response to heat [145]. It was found that copolymers of NIPAM with octadecyl acrylate (ODA) can sensitize non-TSLs to heat or increase the sensitivity and response of TSLs at elevated temperature. Since then NIPAM based copolymers and a variety of synthetic copolymers of *N*-(2-hydroxypropyl) methacrylamide (HPMA) [146,147,148], Poly(acryloylpyrrolidine) (APr) [149,150], (2-ethoxy)ethoxyethyl vinyl ether (EOEOVE) [151,152,153,154], as well as biopolymers such as elastin like peptide (ELP) [138,155,156] and zipper peptide [157,158], have been exploited to prepare temperature-sensitive-polymer modified liposomes (TSPLs).

In contrast to thermosensitive polymeric nanoparticles that exhibit a slow rate of drug release in response to heat, TSPLs, in which high amount of free form of drug is encapsulated inside the liposomal reservoir, exhibit faster and more efficient drug release at hyperthermia, which is comparable to what is observed with TSLs. Besides, compared to TSLs, TSPLs exhibit an additional thermal controlled function which is temperature triggered cell interaction [152,159]. Additionally, presence of hydrophilic polymers on the outer surface of liposomes, at temperatures below the LCST of the polymer, is also expected to stabilize the liposome through the steric hindrance effect of hydrated polymer chains both during storage and also upon exposure to the biological milieu. These make TSPLs great candidates for delivering drugs to tumor cells. However, addition of another functional compartment into the liposome vehicle, adds more complexity to the delivery system

In order to decorate liposomes with TSP, the hydrophilic TSP requires a lipophilic unit or units to be incorporated into the hydrophobic core of the lipid membrane and anchor the polymer in the lipid bilayer (Figure 2c). In one approach several anchor units such as ODA [145,160,161], or *N*,*N*-didodecylacrylamide (NDDAM) [162,163] could be copolymerized with the polymer as side chains of the polymeric backbone. In another approach a lipophilic unit such as phospholipid [159], 2-dodecyl-sulfanylthiocarbonylsulfanyl-2-methyl propionic acid (DMP) [164], 2C12 [162,165,166], or cholesterol [147] have been added to the terminal end of the polymer. The hydrophobic modified polymer can then be added to the liposome either through addition to the organic lipid mixture by which both inner and outer membranes of liposomes will contain the thermosensitive polymer (TSP) upon liposome formation, or by post insertion of the copolymer into preformed liposomes by which only the outer surface will be modified. However, Han et al. showed that the hydrophobic isopropyl groups of NIPAM can also fix the polymer in a liposome membrane [167,168]. Klemetsrud et al. [169] used electrostatic interaction for modification of positively charged liposomes containing 10 mol% DPTAP with the negatively charged NIPAM-co-MAA.

The mechanism by which temperature sensitive polymers trigger drug release from liposomes has not been fully elucidated. Moreover, release varies depending on liposome composition. However, it involves loss of hydrophilicity of the extended polymer chain which moves from a hydrated state at temperatures below the polymer LCST, to the shrunken and dehydrated state of the polymer when the environment temperature has risen above the LCST. Such changes in hydrophilicity and conformational changes have been shown to increase liposomes permeability for the encapsulated drug or may also lead to disintegration of the liposome. The latter is observed in liposomes containing high content of helper lipid dioleoylphosphatidylethanolamine (DOPE), in which thermal response of TSP induces transition of the bilayer liposomal phase into a hexagonal phase, leading to a fast and almost complete disruption of the liposome resulting in payload release (Figure 2c).

Early studies on TSPLs using copolymerized pure NIPAM with about 1 mol% ODA showed the capability of NIPAM in promoting drug release from TSLs composed of DPPC, while it failed to induce drug release from EPC liposomes at hyperthermia [145]. Later Kano and coworkers [160] found that NIPAM-ODA on one hand stabilizes liposomes composed of DOPE, which in absence of copolymer cannot form stable liposomes, and on the other hand, sensitizes DOPE liposomes to heat at temperatures close to LCST of the copolymer. However, the LCST of NIPAM at 32 °C by itself is not practically suitable for hyperthermia application, and copolymerization with hydrophobic ODA moieties results in a broadened endotherm centered at around 30 °C and once inserted in the liposome membrane drug release starts at temperatures below 30 °C [145,160]. The same release pattern at temperatures below the hyperthermia condition was also observed when liposomes composed of DOPE:EPC (64:36 mol%) were modified with NIPAM-NDDAM, where about 70% of encapsulated calcein has been released within 5 min incubation at 30 °C [163].

As stated earlier, one solution to adjust the LCST of TSP for hyperthermia treatments at temperatures around 42 °C is copolymerization of TSP with hydrophilic monomers such as acrylamide (AA). In this regard, Kim et al. [161] found that copolymer of NIPAM with ODA and 2.015 or 3.19 mol% AA (P(NIPAM-ODA-AA)) exhibit a sharp coil to globe transition starting at 37 and 43 °C, respectively. Hayashi et al. [164] synthesized a copolymer of NIPAM-NDDAM-AA (86.7:1:12.3 mol%), which exhibit a cloud point—at which the polymer separates from the solution—at 33.8 °C. When the copolymer was incorporated into liposomes composed of DOPE:EPC (60:40 mol%) the thermal-triggered calcein release started at 40 °C and while less than 20% release observed at 35 °C, around 80% of encapsulated calcein was released over 5 min incubation at 45 °C. Han et al. [167] modified DXR loaded cholesterol containing TSLs composed of DPPC, HSPC and Chol, (56:28:17 mol%) with NIPAM-AA (83:17 mol%, LCST ca 40 °C) without using hydrophobic anchor units. Their TSPL released less than 10% DXR during 5 min incubation at 37–38 °C, while 65% release was achieved over 5 min of incubation at 39 °C. In addition to this they found that addition of 3% mPEG 2000-DSPE into TSPLs improves the stability of these liposomes at 37 °C in presence of 50% serum, while this modification does not affect the drug release pattern from the liposomes. Their PEGylated TSPLs showed better therapeutic activity compared to free DXR or TSLs modified with only PEG or NIPAM-AA against B16F10 melanoma tumor model after injection of a DXR dose of 6 mg/kg followed by 10 min hyperthermia at 42 °C applied 1 h after injection [168].

Ta et al. [164] copolymerized NIPAM with pH sensitive propylacrylic acid (PAA) containing a terminal DMP group to act as anchoring unit p(NIPAM-PAA)-DMP. Addition of 9% PAA increases the LCST of NIPAM to 42 °C at pH 6.5 but decreased the LCST to 28 °C at pH 5.0, presenting dual function in response to heat and reduced pH. Post insertion of the copolymer into preformed DXR loaded liposomes composed of DPPC, HSPC, Chol, and mPEG 2000-DSPE (54:27:16:3 mol%) resulted in PEGylated TSPL with less than 10% DXR leakage after 90 min incubation at 37 °C in presence of serum at pH 7.5, but it showed 37% and 58% drug release over 3 min hyperthermia of 42 or 43 °C, respectively, both at pH 7.5. Meanwhile, the PEGylated TSPL exhibited about 55% drug release at 37 °C at a pH of 5 mimicking lysosomal condition. They also observed a superior antitumor efficacy of their DXR-loaded PEGylated-PTSL compared to free DXR and the liposomal counterpart without a thermosensitive polymer upon a single injection of 5 mg/kg DXR into mice bearing B16F10 melanoma tumor followed by a 5 min hyperthermia (43 °C) applied 6 h after the iv injection [170].

Mo et al. [148] increased the LCST of NIPAM by copolymerization with hydrophilic *N*-(2-hydroxypropyl) methacrylamide (HPMA). The copolymer composed of NIPAM:HPMA (94:6 mol%), and equipped with terminal DMP (p(NIPAM-r-HPMA)-DMP), exhibited an LCST of 42 °C and once post inserted into DXR-loaded preformed liposomes composed of DPPC, HSPC, Chol, and mPEG2000 (55:25:15:3 mol) in a polymer/lipid mass ratio of 1%, a sharp release of about 70% DXR within 1 min was observed, while less than 20% drug leakage occurred after 60 min incubation at 37 °C. They also showed that the burst release of DXR at hyperthermia resulted in deep penetration of DXR in vitro in tumor spheroids and in vivo in mouse tumors.

Copolymerization of NIPAM with Poly(acryloylpyrrolidine) (Apr), which exhibits transition behavior at temperatures around 50 °C, was also found beneficial to adjust the transition temperature of thermosensitive polymers (TSP) [162].

Kono and coworkers [150,162] modified calcein loaded DOPE liposomes with p(Apr-NIPAM)-2C12 (Apr:NIPAM, 81.6:18.4 mol%) and achieved 70% calcein release within 1 min at 42 °C, while less than 20% release was observed during 15 min incubation at 37 °C.

Another TSP with promising results in triggering release from liposomes is (2-ethoxy)ethoxyethyl vinyl ether (EOEOVE), which like NIPAM presents tunable LCST by copolymerization with hydrophilic (increase) or hydrophobic (decrease) comonomers, and was found more effective than NIPAM in sensitizing liposomes to heat [151]. Kono et al. [152] decorated non-thermoresponsive liposomes composed of EYPC:Chol (50:45 mol/mol) with 4 mol% mPEG5000-DSPE and 2 mol% copolymer of EOEOVE-ODVE (MW ca. 18 K) and remotely loaded this nanoparticle with DXR. The particle exhibited minor leakage at 37 °C, 50% release at 43 °C, and 90% release at 45 °C after 3 min incubation at the indicated temperatures in serum free buffer.

In an extensive in vivo study, they found that these DXR-loaded TSPL are more effective than the liposomal counterpart lacking polymers against a murine c-26 tumor when 10 min hyperthermia of 45 °C was applied 6 h after injection [152]. They also showed that the maximum antitumor effect could be obtained when hyperthermia was applied at the moment that intratumoral extravasation-based accumulation of liposomes inside tumor reaches the maximum value [152,153]. For example, application of hyperthermia at 3 h post injection was less effective than hyperthermia at 12 h [152].

HPMA polymers are water soluble, biodegradable, and biocompatible polymers that have been extensively used as macromolecular drug carriers due to their long circulation in blood. Hennink and coworkers found that the LCST of HPMA polymers could be tuned by copolymerization of HPMA-monolactate and HPMA-dilactate [171], and investigated the capability of p(HPMA mono/dilactate) equipped with a cholesterol molecule as anchor, in sensitizing liposomes to heat [146,147]. They found a significant difference between the transition behavior of HPMA copolymers in solution compared to what was observed when incorporated on lipid membranes. Their optimized copolymer (Mw of 10.0 kDa), which was suitable for temperature triggered drug release, was composed of monolactate/dilactate ratio of 44/56, and the copolymer solution exhibited a cloud point of 19 °C. However, when 5 mol% of copolymer was incorporated into liposomes composed of DOPE:EPC (70:25 mol/mol) the TSPL remained stable at 37 °C, while after 5 min exposure to HIFU-induced hyperthermia of 45 °C around 70% of encapsulated DXR was released [146].

In addition to the synthetic polymers, biopolymers have also attracted considerable attention for drug delivery applications [122]. Among these, elastin like polypeptide was found responsive to heat in a narrow temperature window of less than 2 °C [172]. Park et al. [156] synthesized a peptide composed of three repeats of VPGVG amino acids, conjugated to a stearyl group (C18) at *N*-terminus, and amidized the *C*-terminal (SA-ELP3-NH2). Incorporation of 1 mol% of this lipopeptide into liposomes composed of DPPC:Chol:mPEG2000-DSPE (76:21:3 mol%) and loaded with DXR resulted in a fast drug release at mild hyperthermia of 42 °C with ≥95% release within 10 s in the presence of 20% serum, comparable to what was observed with LTSL composed of DPPC:DMPC:mPEG2000-DSPE (90:10:4 mol%). However, compared to LTSL these nanocarriers showed 3-times more leakage stability at 37 °C, exhibited 2-times longer blood circulation half-life after single i.v. injection of 5 mg DXR/kg into BALB/c mice, and finally showed greater antitumor response against a SCC-7 tumor model at a dose of 5 mg DXR/kg when 1 h hyperthermia of 43 °C was applied after the injection.

As stated earlier, addition of functional copolymers into liposomes adds more complexity to the delivery system. So far, a variety of liposomes with different lipid composition have been modified with a variety of copolymers with different chemistry. In fact, most of these studies mainly focused on proving the concept or applicability of such designed system rather than on formulation optimization in order to improve therapeutic activity and performance. Lack of such experimental evidence makes it impossible to discuss in-depth about TSPL, or to correlate or compare the activity of different preparations. Here we provide some insights in the concept, mechanism of drug release, methods of liposome modification, and rationale of copolymer modifications. Additionally, different TSPLs have been introduced concisely while the most critical features of these studies, including formulation details, experimental setting and results, are presented.

## 6. Conclusions

A variety of nanoparticles with thermally triggered drug release profile have been produced and studied in different preclinical settings. Different from polymeric nanoparticles, which require impractical long duration of heating to release significant amount of drug, liposome-based temperature sensitive nanoparticles show more promising results. So far, the most advanced systems are thermosensitive liposomes, with super-fast drug release that should reach complete drug release during passage through the heated area (i.e., the tumor). Next to the capabilities of nanoparticles to deliver and release a payload at the heated area, timing, duration, and degree of hyperthermia are also crucially important parameters. Lessons learned during almost four decades, and more importantly failure of clinical trials, reveal that this combination could be successful when mild hyperthermia (40–42 °C) is applied for 30–60 min (when blood level of nanoparticles is still high) started concomitantly or prior to nanoparticle injection. While temperatures in the range of 40–42 °C are tolerated well by patients, application of higher temperatures (≥43 °C) not only results in patient discomfort but also hampers drug delivery due to the possibility of vascular collapse. Therefore, it is important to optimize temperature sensitive nanoparticle with regards to these conditions. For example, one may think it is possible to adjust heat deposition to a safe thermal dose while using a high temperature, which would be necessary for fast and complete release from nanoparticles acting at a temperature above 42 °C, by decreasing the hyperthermia duration. However, in such setting, in an intravascular drug release treatment, the overall drug delivery is still impaired because only a portion of the administered nanoparticles are being exposed to heat. If extravascular drug release is aimed, it is not rational to overheat the tumor as fast drug release from extravasated nanoparticles is not really beneficial whereas a sustained drug release may likely favor intratumoral drug distribution

## Figures and Tables

**Table 1:** Temperature sensitive polymeric nanoparticles fabricated with polymers exhibiting lower critical solution temperature (LCST).

Copolymer	Drug	Size (nm)	LCST	Release Rate	Ref
PNIPAAm-PBMA poly(*N*-isopropylacrylamide-b-butylmethacrylate)	Doxorubicin	338 ± 23	32.5 °C	57% after 5 h at 37 °C 80% after 5 h at 40 °C	[130]
*P*-(NIPAAm-co-DMAAm)-b-*P*-(d,l-lactide) poly(*N*-isopropylacrylamide-co-*N*,*N*-dimethylacrylamide)-b-poly(d,l-lactide)	Doxorubicin	69.2	40 °C	2.5% after 4 days at 37 °C 11% after 4 days at 42.5 °C	[139]
PNIPAAm-b-PMMA poly(*N*-isopropylacrylamide-b-methyl methacrylate)	Prednisone acetate	190	33 °C	22.5% after 5 h at 27 °C 37 °C na 50% after 5 h at 40 °C	[131]
*P*-(NIPAAm-co-AAm)-b-PDLLA poly(*N*-isopropylacrylamide-co-acrylamide)-b-poly(d,l-lactide)	Docetaxel	80	40 °C	30% after 10 h at 37 °C 36% after 10 h at 43 °C	[140]
*P*-(NIPAm-co-AAm) poly(*N*-isopropylacrylamide-co-acrylamide)	Docetaxel	50	40 °C	80% after 50 h at 37 °C 40% after 50 h at 43 °C	[126]
*P*-(NIPAAm-co-AAm)-b-PBMA poly(*N*-isopropylacrylamide-co-acrylamide)-b-poly(n-butyl methacrylate)	Methotrexate	175 ± 15	40 °C	21% after 5 h at 37 °C 53% after 5 h at 42 °C	[141]
*P*-(NIPAAm-co-NHMAAm)-b-PCL *P*-(*N*,*N*-isopropylacrylamide-co-*N*-hydroxymethylacrylamide)-b-caprolactone	Doxorubicin	97.2 ± 20.4	38 °C	35% after 25 h at 38 °C Na at 37 57% after 25 h at 43 °C	[128]
PNI-U-DPy poly(*N*-isopropylacrylamide) containing pendant U-DPy	Doxorubicin	164	34 °C	63% 2.5 h 37	[133]
Cy-functionalized supramolecular polymer, Cy-PPG	Doxorubicin	76	45 °C	5% after 2.5 h at 25 °C 37 °C na 85% after 2.5 h at 40 °C	[121]
NIPAM-co-AAc poly(NIPAM-co-acrylic acid)	Doxorubicin retinoic acid	400 at 25 °C 100 at 37 °C	37.2 °C	40% after 4 h at 37 °C 80% after 48 h at 37 °C	[127]
Pentaerythritol polycaprolactone-b-poly(*N*-isopropylacrylamide)-folic acid (four star-arm PE-PCL-b-PNIPAM-FA)	Doxorubicin	85	31 °C	5% after 5 h at 37 °C 45% after 5 h at 40 °C	[142]
Pentaerythritol polycaprolactone-b-poly(*N*-vinylcaprolactam)-Folic acid (four star-arm PE-PCL-b-PNVCL-FA)	Doxorubicin	185	39 °C	5% after 5 h at 37 °C 50% after 5 h at 40 °C	[142]
(cb-*P*-(HEMA-g-*P*-(NIPAAm-st-HEAAm))) cyclic brush poly(2-hydroxyethyl methacrylate-gpoly(*N*-isopropylacrylamide-st-*N*-hydroxyethylacrylamide)) (cb-*P*-(HEMA-g-*P*-(NIPAAm-st-HEAAm)))	Doxorubicin	28	38 °C	28% after 40 h at 25 °C 32% after 40 h at 37 °C 52.5% after 40 h at 40 °C	[120]
